# Characterization and Tentative Annotation of Flavonoid Glycosides in a Flavonoid-Enriched Fraction from *Bambusa textilis* Leaves by UHPLC-ESI-Q-TOF-MS/MS

**DOI:** 10.3390/plants15162459

**Published:** 2026-08-13

**Authors:** Yuan Fang, Ting Yuan, Xuefeng Guo

**Affiliations:** Key Laboratory of National Forestry and Grassland Administration on Bamboo & Rattan Science and Technology, International Center for Bamboo & Rattan, Beijing 100102, China; fang87606@163.com (Y.F.); yuanting_jm@163.com (T.Y.)

**Keywords:** *Bambusa textilis*, flavonoid-enriched fraction, flavonoid glycosides, UHPLC-ESI-Q-TOF-MS/MS, tentative annotation, fragmentation pathways

## Abstract

Bamboo leaves contain structurally diverse flavonoid glycosides; whereas, compound-level information for *Bambusa textilis* remains limited. In this study, a flavonoid-enriched 40% ethanol fraction prepared from *B. textilis* leaves by petroleum-ether partitioning and AB-8 resin adsorption was analyzed by ultra-high-performance liquid chromatography coupled with electrospray ionization quadrupole time-of-flight tandem mass spectrometry (UHPLC-ESI-Q-TOF-MS/MS) in negative-ion mode. Retention times and MS/MS spectra were compared with seven in-house reference standards, while the remaining constituents were annotated from accurate-mass measurements, diagnostic product ions, fragmentation behavior, and data from the literature. According to Metabolomics Standards Initiative criteria, seven compounds were assigned at Level 1 and twenty-four were reported as Level 3 annotations because their exact positional structures were not confirmed by additional standards or NMR. Thirty-one flavonoid-related constituents were annotated in the analyzed fraction, encompassing six glycoside classes; most annotations, by count, were apigenin- or luteolin-derived. These annotations add compound-level information for the analyzed *B. textilis* fraction and allow qualitative comparison with other bamboo flavonoid profiles. Because the sample was selectively enriched, the results do not describe the complete leaf metabolome or compound concentrations. Additional standards or NMR analysis are needed to confirm the glycosylation positions of tentatively annotated compounds.

## 1. Introduction

Bamboo leaves are natural plant materials with long-standing food and medicinal uses. Flavonoids are among the principal bioactive constituents of bamboo leaf extracts [1,2], and bamboo leaf extract has also been used in products related to natural food additives [3]. Accordingly, knowledge of the flavonoid composition and structural diversity of bamboo leaves is important for the rational utilization and quality evaluation of this resource.

*Bambusa textilis* McClure is an important sympodial bamboo species of the genus Bambusa. Three antioxidant flavone glycosides have previously been isolated from its leaves [4], while broader studies have mainly addressed active fractions or total flavonoid content [5,6]. Compound-level information across the different flavonoid glycoside subclasses in *B. textilis* leaves remains limited. Conventional isolation followed by nuclear magnetic resonance spectroscopy provides definitive structural information but requires relatively large amounts of material and substantial time, which limits its use for rapid profiling of complex extracts.

High-performance liquid chromatography coupled with high-resolution mass spectrometry has been used to characterize flavonoids in leaves of *Phyllostachys edulis*, *Bambusa chungii*, and *Pleioblastus maculatus* [7,8,9]. More recently, LC-ESI-QTOF-MS/MS profiling across eleven bamboo leaf varieties provisionally annotated 81 phenolic compounds and again showed a predominance of luteolin- and apigenin-derived flavones [10]. Untargeted LC-QTOF metabolomics across twelve bamboo species further demonstrated substantial interspecific and environmentally associated variation in phenolic profiles [11]. Recent reviews and multi-species studies likewise emphasize the chemical diversity of bamboo leaves and the effects of species and growth stage on their measured phytochemical profiles [12,13]. The reported variation among species also means that analytical scope and annotation confidence are important when comparing bamboo profiles.

UHPLC-ESI-Q-TOF-MS combines efficient chromatographic separation with accurate-mass measurement and sensitive tandem-mass detection and has been applied to phenolic and flavonoid profiling in complex plant extracts [14,15,16,17]. In this study, flavonoid glycosides in the 40% ethanol, resin-enriched fraction of *B. textilis* leaves were examined by UHPLC-ESI-Q-TOF-MS/MS in negative-ion mode, with particular attention to diagnostic product ions and fragmentation behavior. The analysis was qualitative and specific to this fraction, and standard-confirmed identifications were reported separately from tentative annotations based on mass-spectral evidence.

## 2. Results

### 2.1. UHPLC-Q-TOF-MS Analysis of the Flavonoid-Enriched Fraction

**Figure 1:**
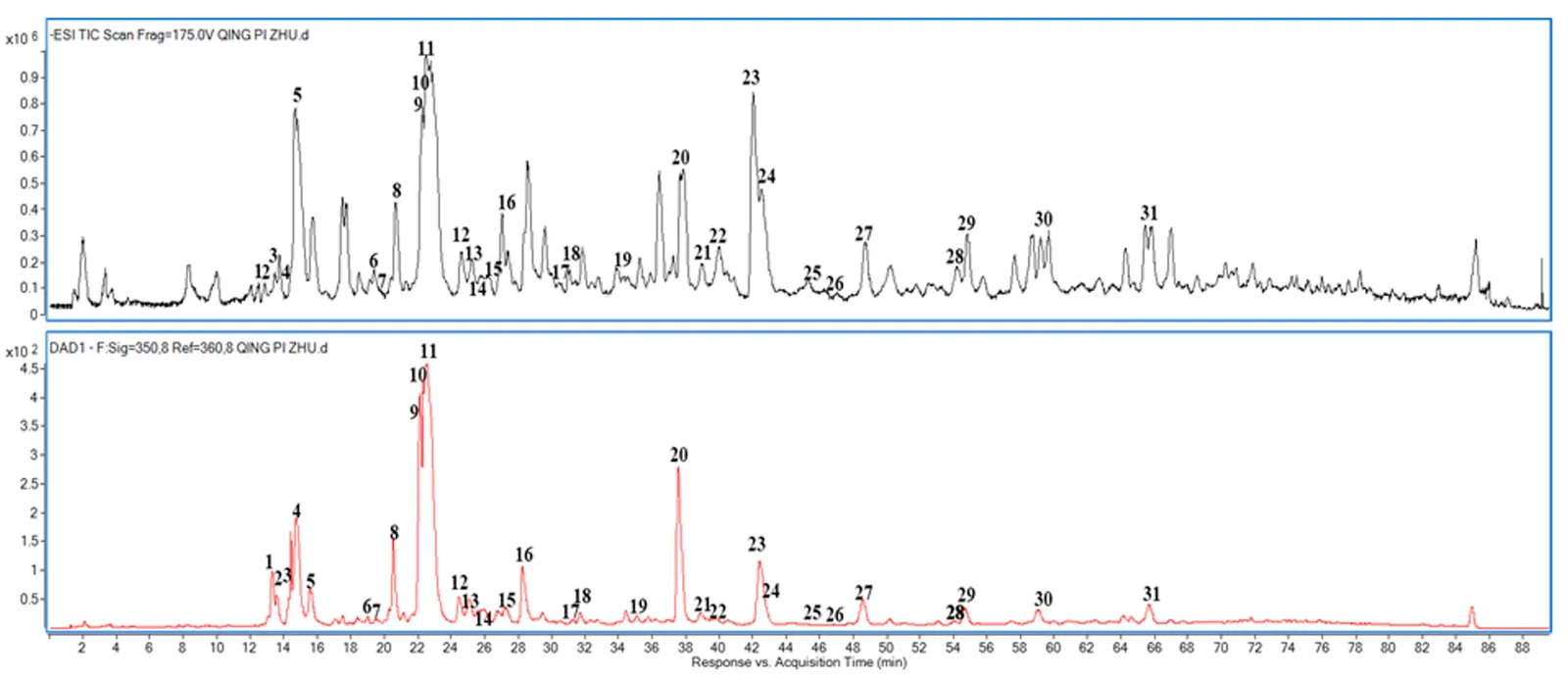
UHPLC-UV chromatogram acquired at 350 nm and total ion chromatogram of the flavonoid-enriched fraction from *Bambusa textilis* leaves.

**Figure 2:**
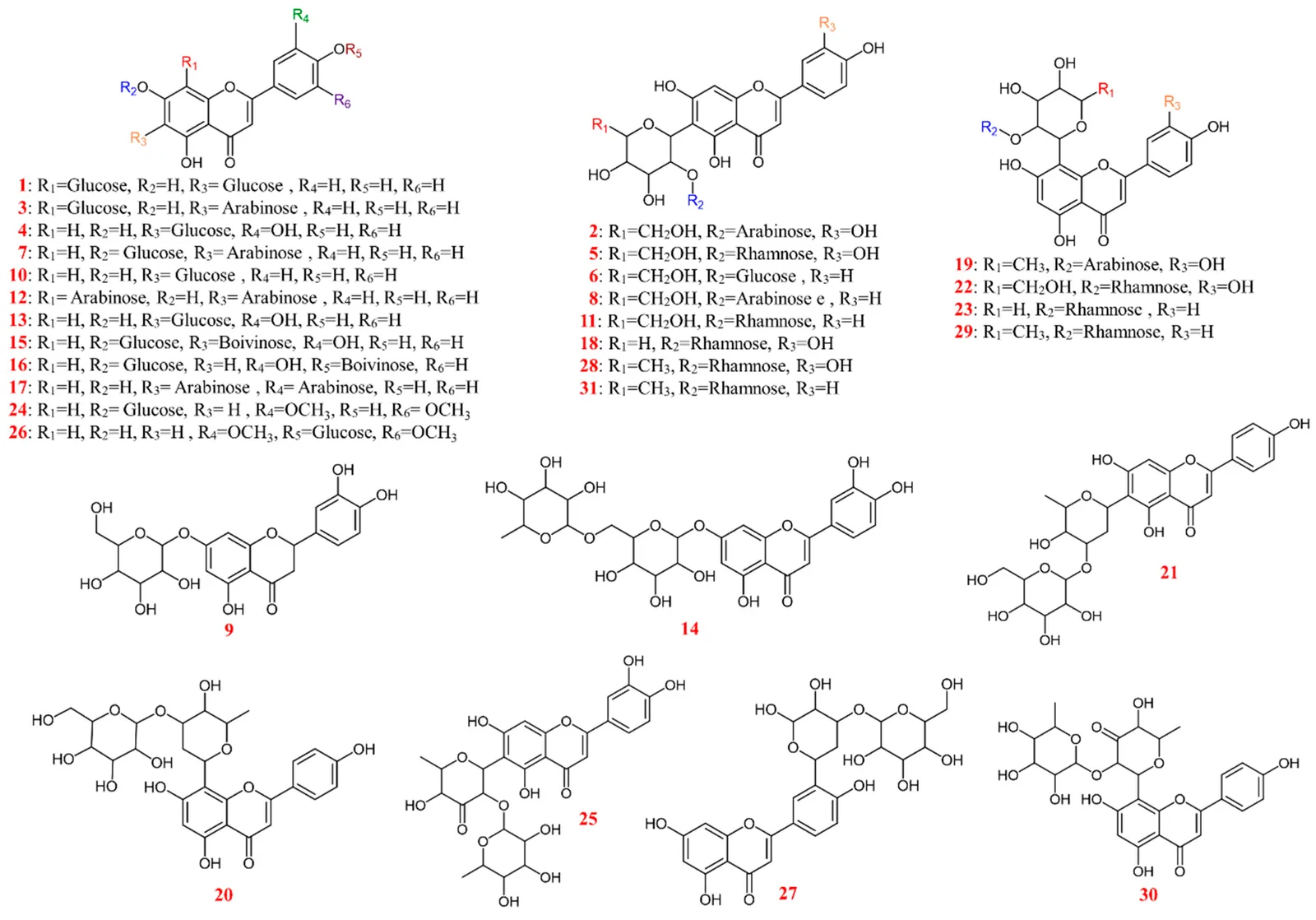
Candidate flavonoid structures in the *Bambusa textilis* leaf fraction; positions not confirmed by authentic standards are provisional. Colors distinguish sites and compound numbers only.

The UHPLC-UV chromatogram acquired at 350 nm and the total ion chromatogram obtained by UHPLC-ESI-Q-TOF-MS/MS are shown in Figure 1. Thirty-one flavonoid-related constituents were detected and annotated in the analyzed *B. textilis* leaf fraction. Seven compounds were assigned by comparison of retention times and MS/MS spectra with authentic in-house reference standards and were classified as Metabolomics Standards Initiative (MSI) Level 1 identifications. The remaining twenty-four compounds were annotated from accurate-mass data, diagnostic product ions, established fragmentation nomenclature [18,19], and published mass-spectral information; because exact positional structures were not confirmed by additional standards or NMR, these entries were conservatively classified as MSI Level 3 annotations [20]. Retention times, molecular formulas, calculated and measured precursor ions, mass errors, product ions, annotations, and confidence levels are summarized in Table 1. The candidate structures shown in Figure 2 represent working annotations rather than definitive structures for compounds without standards.

The analysis was qualitative; no validated chromatographic quantification or formal determination of chromatographic resolution (Rs) was performed. Potential co-elution therefore cannot be excluded. Accurate precursor masses and compound-specific product-ion spectra supported the annotations but did not establish baseline chromatographic separation. The relative ion intensities in Table 1 represent spectral responses rather than compound concentrations in the leaf fraction.

### 2.2. Flavone C-Glycosides

**Figure 3:**
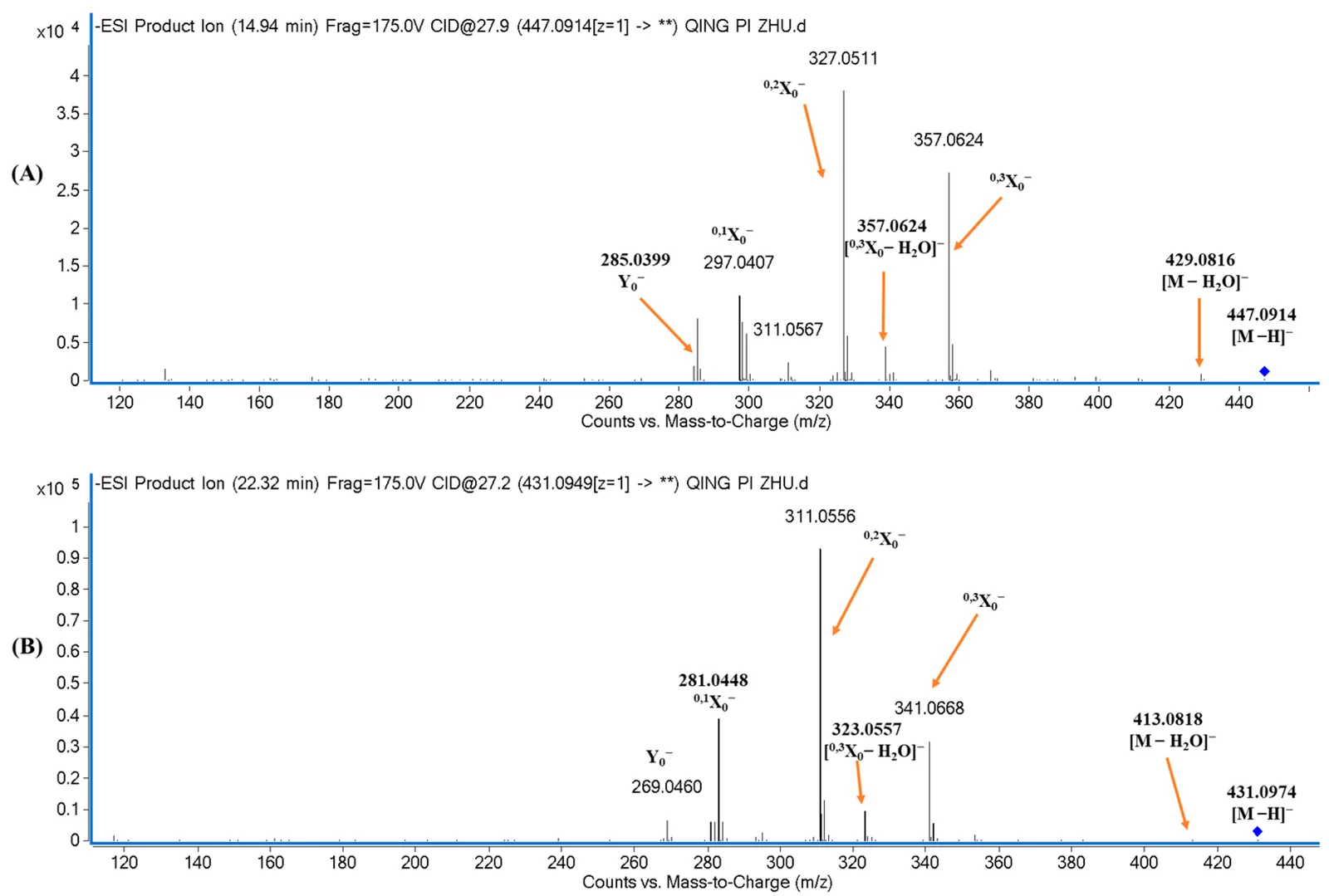
Product-ion spectra of the deprotonated ions [M-H]^−^ of compounds **4** and **10**: (**A**) compound **4**; (**B**) compound **10**. Here, “**” means all detected product ions (not statistical significance).

Compounds **4** and **10** had molecular formulas C_21_H_20_O_11_ and C_21_H_20_O_10_ and retention times of 14.94 and 22.32 min, respectively. Comparison with authentic standards identified compound **4** as luteolin 6-C-glucoside and compound **10** as apigenin 6-C-glucoside. Their product-ion spectra are shown in Figure 3A,B.

Compound **4** produced the deprotonated ion [M-H]^−^ at *m*/*z* 447.0914. The product ion at *m*/*z* 285.0412 [Y0]^−^ corresponded to the luteolin aglycone after loss of a hexosyl moiety (162 Da), and the ion at *m*/*z* 133.0308 [1,3B0]^−^ was generated by retro-Diels–Alder (RDA) cleavage. Product ions at *m*/*z* 357.0624 (0,3X0), 327.0511 (0,2X0), and 297.0407 (0,1X0) reflected neutral losses of 90, 120, and 150 Da from the C-linked glucose unit, respectively. Ions at *m*/*z* 429.0816 [M-H-H2O]^−^ and 339.0503 [0,3X0-H2O]^−^ arose from dehydration of the precursor and cross-ring-cleavage ions.

Compound **10** yielded [M-H]^−^ at *m*/*z* 431.0974 and a dehydration ion at *m*/*z* 413.0818. The series at *m*/*z* 341.0668 (0,3X0), 311.0556 (0,2X0), and 281.0448 (0,1X0) resulted from neutral losses of 90, 120, and 150 Da, which are characteristic of C-glycoside cross-ring cleavage. The ion at *m*/*z* 323.0554 was assigned as [0,3X0-H2O]^−^, while *m*/*z* 269.0460 [Y0]^−^ and 117.0339 [1,3B0]^−^ supported an apigenin aglycone. As expected, the corresponding product ions were 16 Da lower than those of the luteolin derivative, compound **4**.

### 2.3. Flavone O-Glycosides

**Figure 4:**
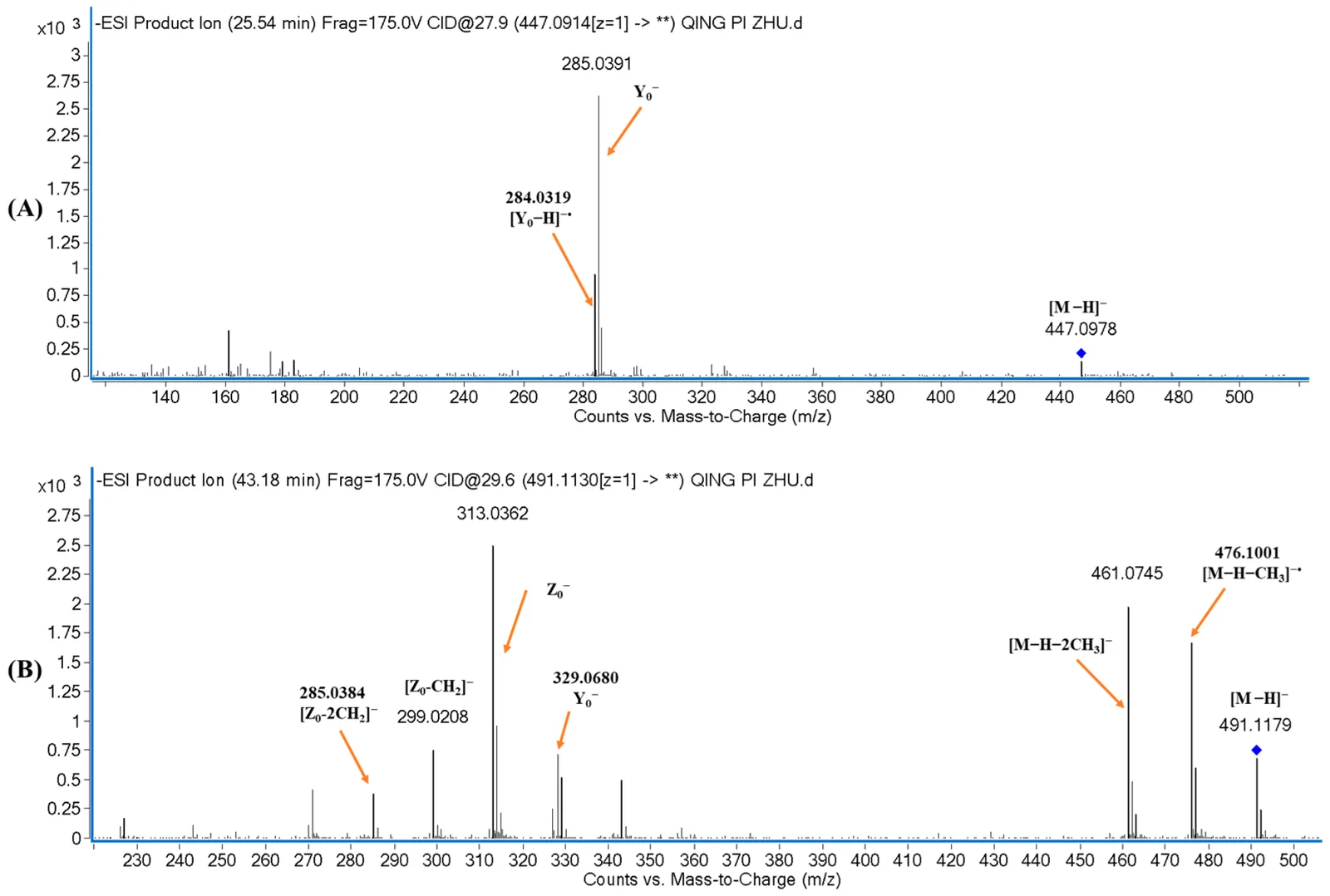
Product-ion spectra of the deprotonated ions [M-H]^−^ of compounds **13** and **24**: (**A**) compound **13**; (**B**) compound **24**. Here, “**” means all detected product ions (not statistical significance).

Compounds **13** and **24** yielded deprotonated ions at *m*/*z* 447.0914 and 491.1179 and had molecular formulas C_21_H_20_O_11_ and C_23_H_24_O_12_, respectively. Comparison with reference standards identified compound **13** as luteolin 7-O-glucoside (tR = 25.31 min) and compound **24** as tricin 7-O-glucoside (tR = 43.18 min). Their product-ion spectra are presented in Figure 4A,B.

**Figure 5:**
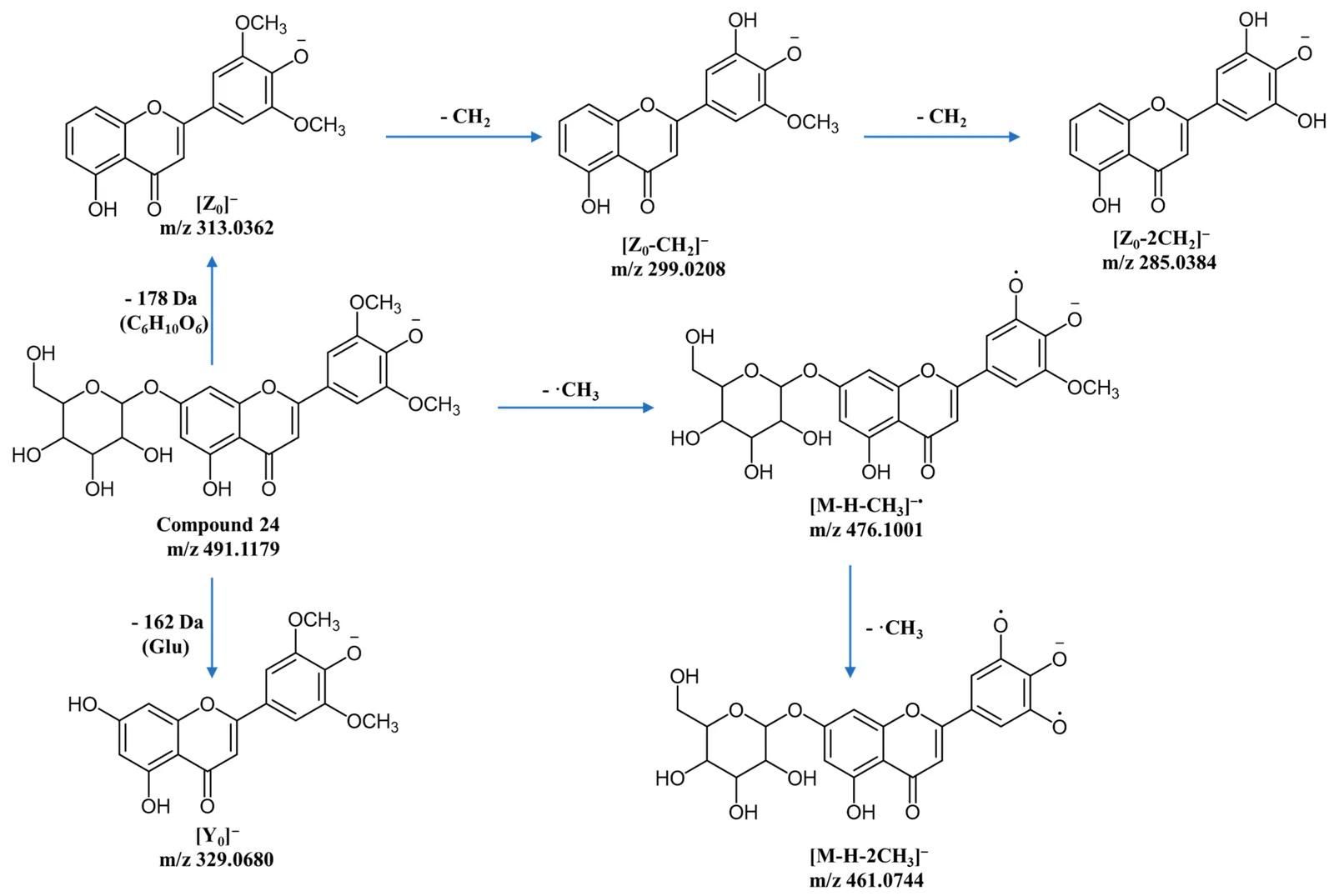
Proposed principal fragmentation pathway of the deprotonated ion [M-H]^−^ of compound **24**.

For compound **13**, direct loss of an O-linked hexosyl moiety (162 Da) from [M-H]^−^ generated the abundant ion at *m*/*z* 285.0391 [Y0]^−^. The radical aglycone ion at *m*/*z* 284.0319 [Y0-H]^−^ was also prominent, and RDA fragmentation produced ions at *m*/*z* 151.0041 [1,3A0]^−^ and 133.0323 [1,3B0]^−^ [21,22]. In contrast, tricin contains methoxy substituents at C-3′ and C-5′. These groups enable sequential methyl-radical losses, accounting for the characteristic ions of compound **24** at *m*/*z* 476.0982 [M-H-CH3]^−^ and 461.0745 [M-H-2CH3]^−^. Loss of O-linked glucose yielded *m*/*z* 329.0680 [Y0]^−^, followed by the diagnostic ion at *m*/*z* 313.0362 and further 14-Da losses to *m*/*z* 299.0208 and 285.0384. Luteolin lacks these methoxy groups; consequently, its negative-ion spectrum is dominated by glycosidic cleavage and RDA products rather than sequential methyl losses. This difference supports aglycone-class assignment but does not, in the absence of a standard, establish an exact O-glycosylation position. The proposed principal fragmentation pathway of compound **24** is shown in Figure 5.

Compound **9** (tR = 21.93 min; C_21_H_22_O_11_) gave [M-H]^−^ at *m*/*z* 449.1064 and an aglycone ion at *m*/*z* 287.0571 after loss of 162 Da. RDA product ions at *m*/*z* 151.0028 and 135.0440 supported an eriodictyol core; it was therefore annotated as an eriodictyol O-glucoside positional isomer. Compound **26** (tR = 47.91 min; C_23_H_24_O_12_) was an isomer of compound **24** and generated [M-H]^−^ at *m*/*z* 491.1155, [Y0]^−^ at *m*/*z* 329.0665, and an ion at *m*/*z* 313.0348. Differences in methyl-loss behavior and comparison with published data [23,24] supported its annotation as a tricin O-glucoside positional isomer; the specific attachment position remains unconfirmed.

### 2.4. Flavone Di-C,O-Glycosides

**Figure 6:**
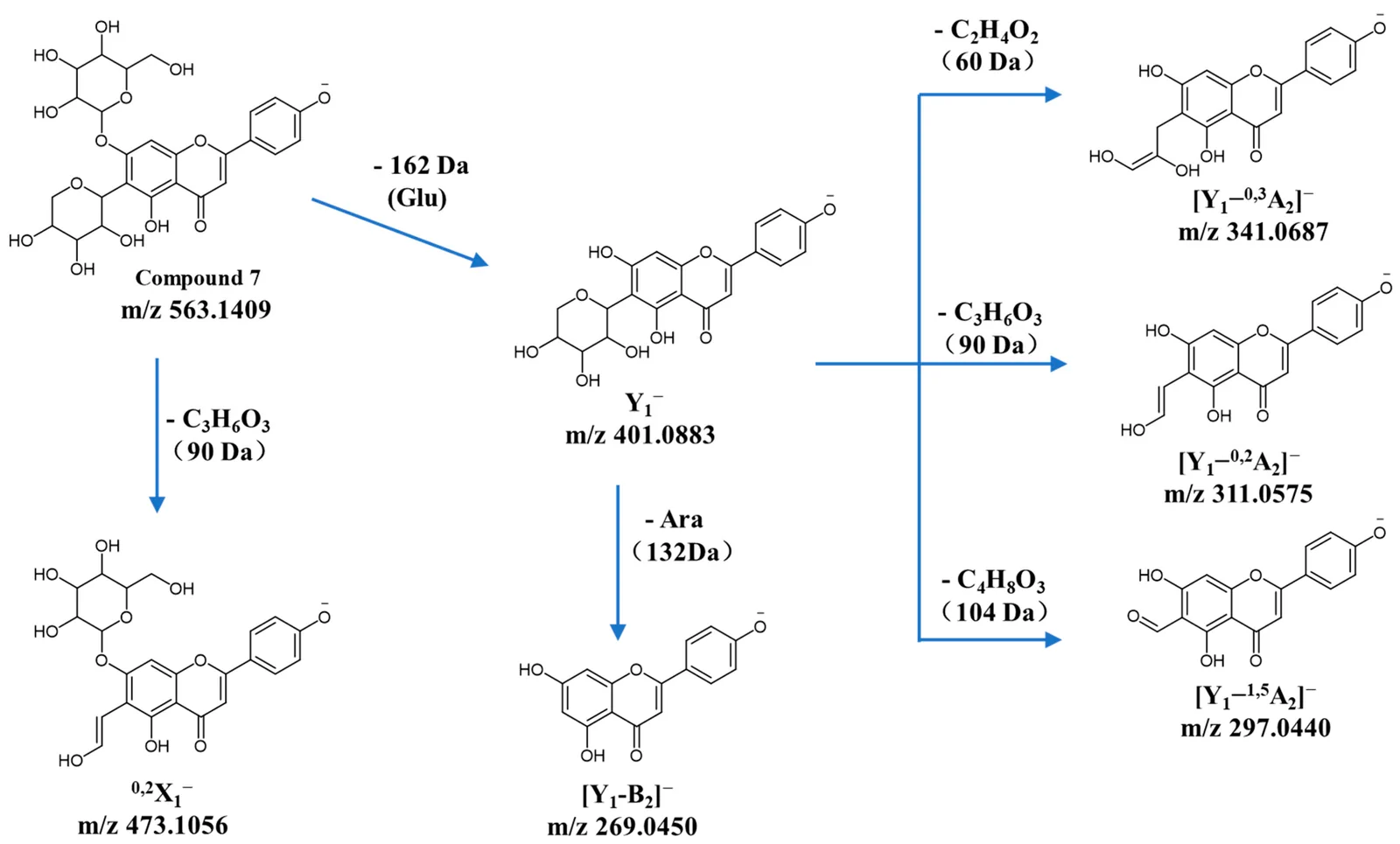
Proposed principal fragmentation pathway of the deprotonated ion [M-H]^−^ of compound **7**.

**Figure 7:**
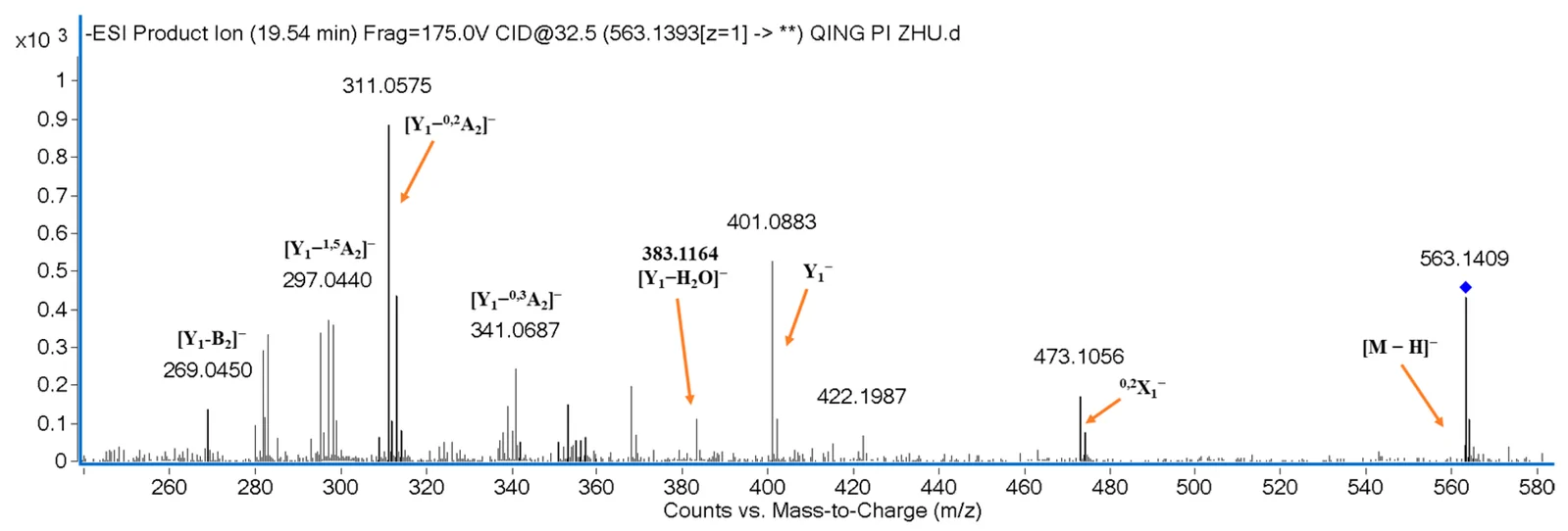
Product-ion spectrum of the deprotonated ion [M-H]^−^ of compound **7**. Here, “**” means all detected product ions (not statistical significance).

Compound **7** (tR = 19.54 min; C_26_H_28_O_14_) was identified as apigenin 6-C-arabinoside 7-O-glucoside by comparison with an authentic standard. The [M-H]^−^ ion at *m*/*z* 563.1409 generated an abundant product ion at *m*/*z* 401.0883 [Y1]^−^ after direct loss of an O-linked glucose unit (162 Da). Product ions at *m*/*z* 473.1056 (0,2X1), 341.0687 [Y1-0,3A2]^−^, 311.0575 [Y1-0,2A2]^−^, and 297.0440 [Y1-1,5A2]^−^ reflected cross-ring cleavage of a C-linked arabinose unit; whereas, *m*/*z* 269.0450 [Y1-B2]^−^ supported the apigenin aglycone. The proposed pathway and product-ion spectrum are shown in Figure 6 and Figure 7.

Compounds **15** and **16** yielded [M-H]^−^ ions at *m*/*z* 577.1551 and 577.1545. Loss of an O-linked glucose produced ions at *m*/*z* 415.1026 and 415.1038 [Y1]^−^, respectively, and the aglycone-related ions near *m*/*z* 285 together with *m*/*z* 133.0306 supported luteolin derivatives. Cross-ring and dehydration products supported a C-linked boivinosyl residue and an O-linked glucosyl residue. Because the two spectra were closely related and no authentic standards were available, compounds **15** and **16** were conservatively annotated as luteolin C-boivinosyl-O-glucoside positional isomers I and II, respectively; their precise attachment positions cannot be established from these data alone.

### 2.5. Flavone Di-C-Glycosides

**Figure 8:**
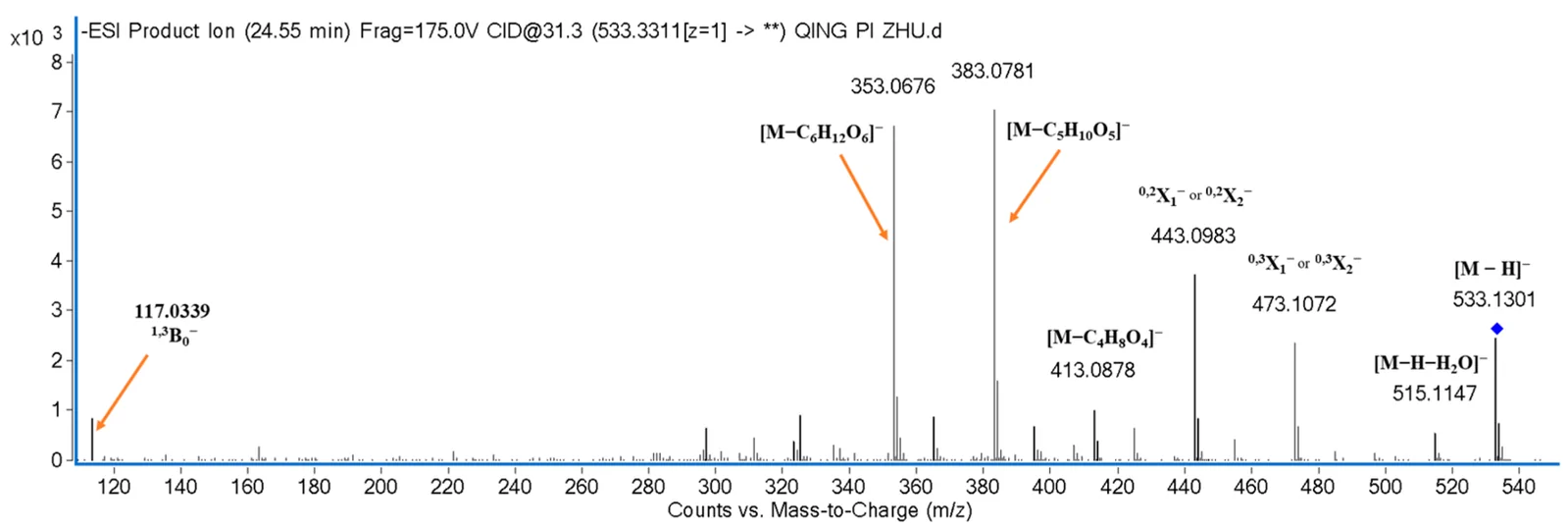
Product-ion spectrum of the deprotonated ion [M-H]^−^ of compound **12**. Here, “**” means all detected product ions (not statistical significance).

Compound **12** (tR = 24.55 min; C_25_H_26_O_13_) was identified as apigenin 6,8-di-C-arabinoside by comparison with a reference standard. Its [M-H]^−^ ion at *m*/*z* 533.1301 generated ions at *m*/*z* 473.1072 and 443.0983 through losses of 60 and 90 Da. Combined cross-ring losses from the two pentosyl substituents produced ions at *m*/*z* 413.0878, 383.0781, and 353.0767, corresponding to total neutral losses of 120, 150, and 180 Da. The particularly abundant ion near *m*/*z* 383 was consistent with two alternative combinations of 60- and 90-Da losses from the C-linked arabinosyl units. The product-ion spectrum is shown in Figure 8.

Compound **3** (tR = 14.36 min; C_26_H_28_O_14_) gave [M-H]^−^ at *m*/*z* 563.1418. Product ions at *m*/*z* 473.1109, 443.0998, 413.0891, 383.0794, 353.0688, and 323.0589 corresponded to successive or combined cross-ring losses. The fragmentation pattern indicated one C-linked arabinosyl and one C-linked glucosyl unit, while *m*/*z* 117.0328 [1,3B0]^−^ supported apigenin as the aglycone. Compound **3** was therefore annotated as an apigenin C-arabinosyl-C-glucoside positional isomer; the two attachment positions remain unresolved.

Compound **17** (tR = 31.32 min; C_25_H_26_O_13_) yielded [M-H]^−^ at *m*/*z* 533.1287 and product ions at *m*/*z* 473.1020, 443.1016, 413.0841, 383.0764, and 353.0655. Its close similarity to compound **12** supported annotation as an apigenin di-C-arabinoside positional isomer. Compound **1** (tR = 13.37 min; C_27_H_30_O_15_) yielded [M-H]^−^ at *m*/*z* 593.1501. Cross-ring losses and aglycone-related ions supported an apigenin derivative bearing two C-linked glucose units; it was annotated as an apigenin di-C-glucoside positional isomer. Exact C-glycosylation positions for compounds **1** and **17** remain unconfirmed.

### 2.6. Flavone C,O-Diglycosides

**Figure 9:**
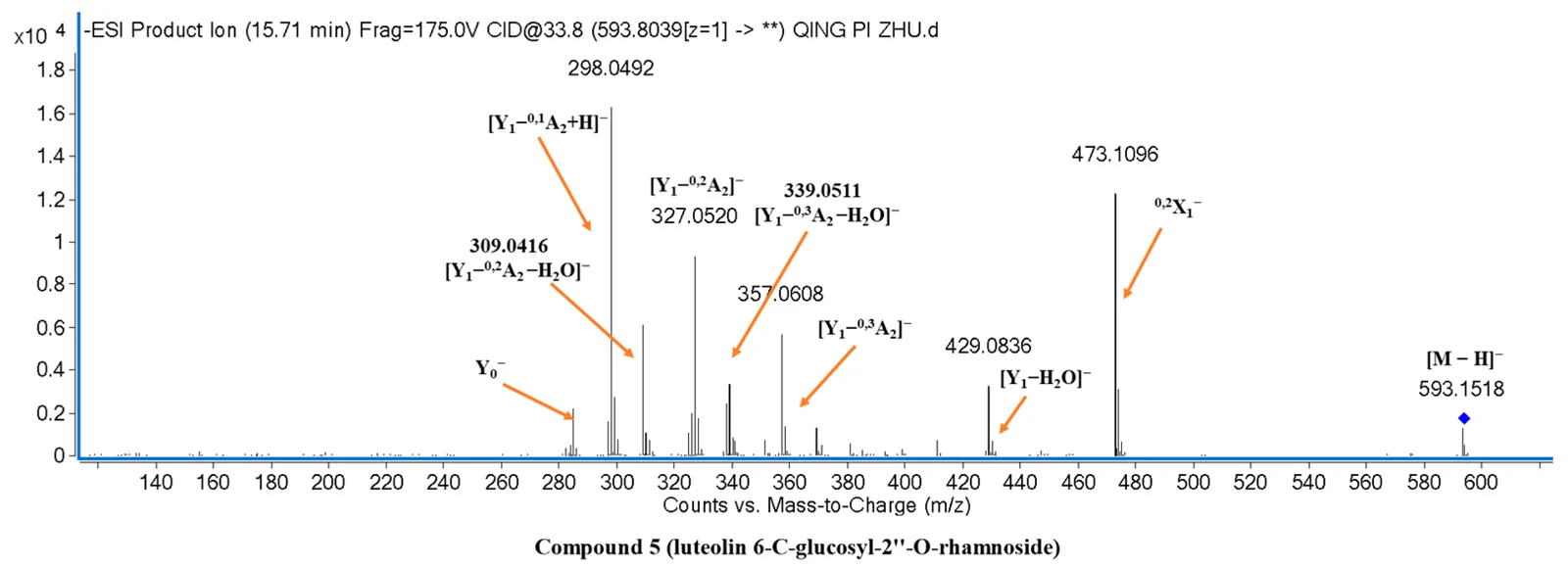
Product-ion spectrum of the deprotonated ion [M-H]^−^ of compound **5**. Here, “**” means all detected product ions (not statistical significance).

**Figure 10:**
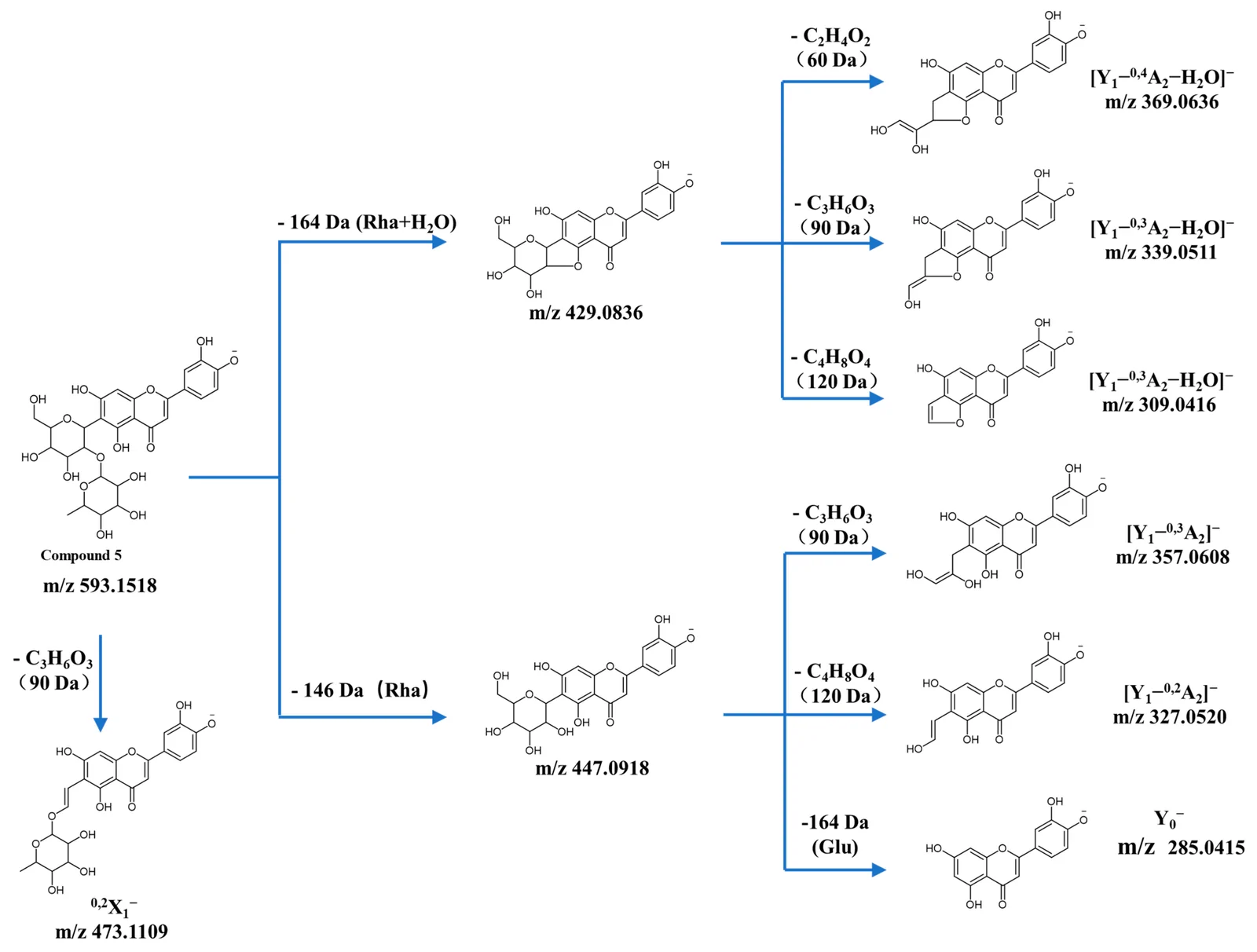
Proposed principal fragmentation pathway of the deprotonated ion [M-H]^−^ of compound **5**.

Compound **5** (tR = 15.71 min; C_27_H_30_O_15_) was identified as luteolin 6-C-(2″-O-rhamnosyl)glucoside by comparison with an authentic standard. It yielded [M-H]^−^ at *m*/*z* 593.1518. Ions at *m*/*z* 285.0415 and 133.0294 [1,3B0]^−^ supported a luteolin aglycone, and the abundant ion at *m*/*z* 473.1096 arose from cross-ring cleavage of the C-linked glucose. Loss of the terminal rhamnosyl unit produced *m*/*z* 447.0918 [Y1]^−^, while the more abundant ion at *m*/*z* 429.0836 [Y1-H2O]^−^ reflected additional dehydration. Further cross-ring ions at *m*/*z* 357.0608, 327.0520, and 298.0492, together with their dehydrated counterparts at *m*/*z* 339.0511 and 309.0416, supported a 6-C-linked glucosyl residue. The product-ion spectrum and proposed pathway are shown in Figure 9 and Figure 10.

Compounds **2**, **6**, **8**, **11**, **18**–**23**, **25**, and **27**–**31** showed the characteristic combination of a low-abundance [Y1]^−^ ion, a more abundant [Y1-H2O]^−^ ion, and cross-ring fragments from a C-linked sugar. These signals support mixed C,O-diglycoside structures, while the exact positions of the C-linked and O-linked residues cannot be assigned without standards. Their retention times, precursor ions, product ions, and conservative annotations are listed in Table 1.

Compounds **2**, **8**, and **11** produced luteolin- or apigenin-related aglycone ions together with fragments diagnostic of C-linked glucosyl and terminal O-linked pentosyl or deoxyhexosyl residues. They were annotated as luteolin C-glucosyl-O-arabinoside positional isomer, apigenin C-glucosyl-O-arabinoside positional isomer, and apigenin C-glucosyl-O-rhamnoside positional isomer, respectively. Compounds **18**, **19**, and **23** were similarly annotated as a luteolin C-arabinosyl-O-rhamnoside positional isomer, a luteolin C-rhamnosyl-O-arabinoside positional isomer, and an apigenin C-arabinosyl-O-rhamnoside positional isomer, respectively [25]. These assignments specify sugar composition and linkage class but not the attachment positions on the aglycone.

Compounds **20**, **21**, and **27** shared the formula C_27_H_30_O_13_ and closely related product-ion spectra, including losses consistent with C-linked boivinose and O-linked glucose. They were therefore annotated as apigenin C-boivinosyl-O-glucoside positional isomers I-III in retention-time order. Retention time and differences in relative product-ion intensities were used only as supporting evidence to distinguish the chromatographic features; they were not considered sufficient to assign specific aglycone attachment positions.

Compounds **22** and **28** (C_27_H_30_O_14_) were annotated as luteolin C-rhamnosyl-O-rhamnoside positional isomers I and II, while compounds **29** and **31** (C_27_H_30_O_13_) were annotated as apigenin C-rhamnosyl-O-rhamnoside positional isomers I and II. Their paired spectra shared precursor formulas and core fragmentation pathways but differed in retention time and relative dehydration-ion abundance. Because such differences are method-dependent and no standards were available, no specific 6-C or 8-C assignment is retained in the revised annotations.

Compound **25** (tR = 45.34 min; C_27_H_28_O_14_) yielded [M-H]^−^ at *m*/*z* 575.1388, with product ions supporting a luteolin C,O-diglycoside containing a dehydropentosyl residue; comparison with published data led to annotation as a cassiaoccidentalin B-type isomer [26]. Compound **30** (tR = 59.35 min; C_27_H_28_O_13_) produced [M-H]^−^ at *m*/*z* 559.1436 and apigenin-related fragments and was annotated as a cassiaoccidentalin A-type isomer. These names denote candidate isomer classes rather than definitive positional structures.

### 2.7. Flavone O,O-Diglycoside

**Figure 11:**
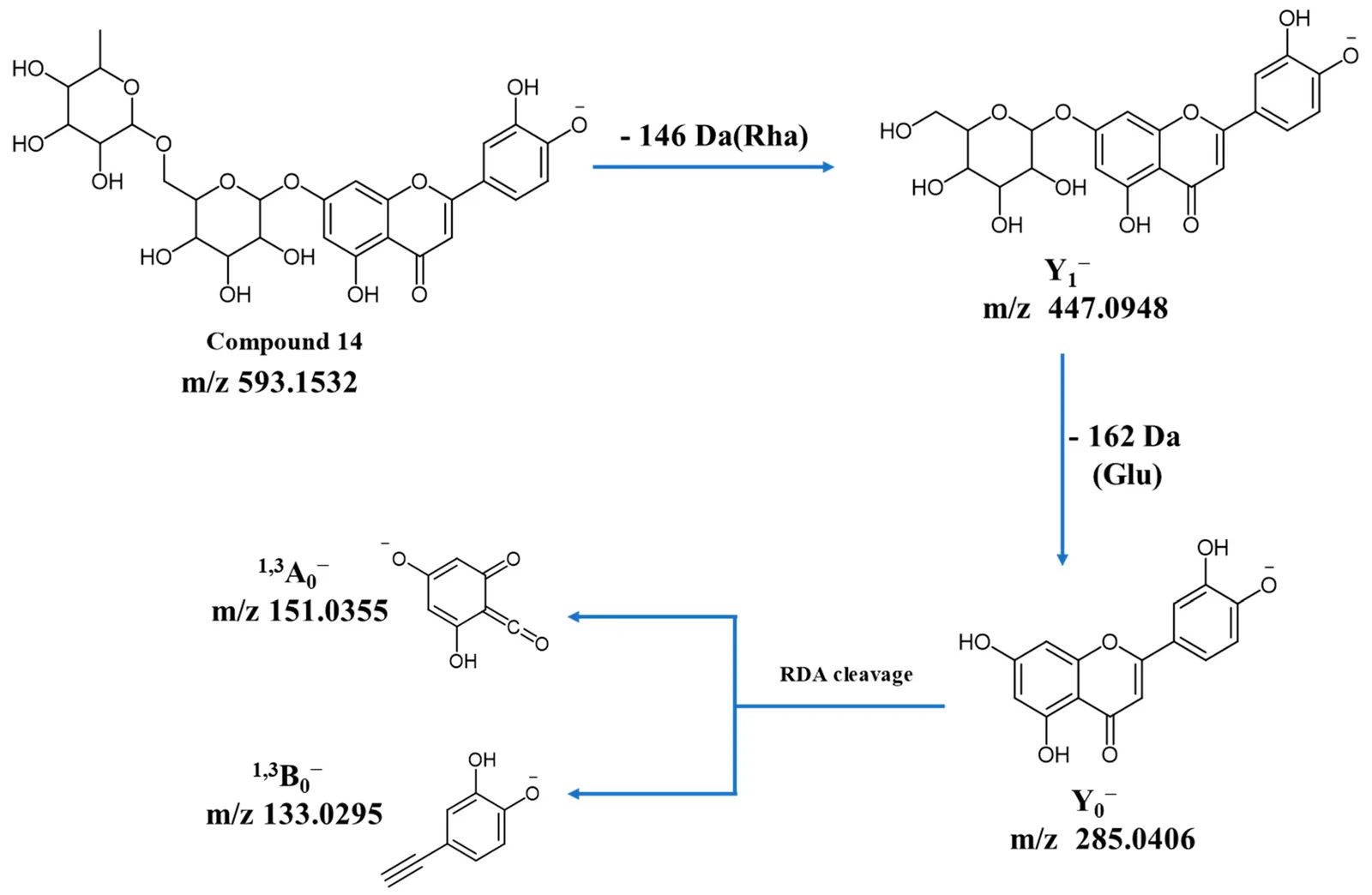
Candidate principal fragmentation pathway of the deprotonated ion [M-H]^−^ of compound **14**. The exact glycosylation positions remain unconfirmed.

Compound **14** (tR = 25.54 min; C_27_H_30_O_15_) yielded [M-H]^−^ at *m*/*z* 593.1532. Sequential losses of an O-linked rhamnosyl unit (146 Da) and an O-linked glucosyl unit (162 Da) generated ions at *m*/*z* 447.0948 [Y1]^−^ and 285.0406 [Y0]^−^, respectively, supporting an O,O-diglycoside with luteolin as the aglycone. RDA ions at *m*/*z* 151.0355 and 133.0295 further supported the luteolin core. Compound **14** was conservatively annotated as a luteolin O-glucosyl-O-rhamnoside positional isomer; its exact aglycone attachment position and interglycosidic linkage position remain unconfirmed. The candidate fragmentation pathway is shown in Figure 11.

## 3. Discussion

### 3.1. Diagnostic Fragmentation of Flavonoid Glycosides

The negative-ion MS/MS data showed class-dependent fragmentation behavior consistent with previous bamboo-leaf studies [7,8,9,10]. O-Glycosides preferentially lost intact glycosyl residues and generated abundant aglycone ions; whereas, C-glycosides underwent cross-ring cleavage of the C-linked sugar. Mixed C,O-glycosides combined direct loss of the terminal O-linked residue with cross-ring fragmentation of the remaining C-linked sugar. RDA cleavage provided complementary evidence for apigenin-, luteolin-, tricin-, and eriodictyol-related aglycones. These patterns are valuable for assigning glycoside class and sugar composition. However, relative dehydration-ion abundance and chromatographic order can vary with collision energy, sugar identity, matrix, and analytical conditions; they therefore provide positional hypotheses rather than definitive evidence for aglycone attachment sites.

### 3.2. Flavonoid Profile of Bambusa textilis Leaves

The analyzed *B. textilis* fraction contained annotations spanning six glycoside classes. By annotation count, apigenin- and luteolin-related structures were most frequent, while fewer annotations were assigned to tricin- and eriodictyol-related constituents. This pattern is broadly consistent with reports for *P. edulis*, *B. chungii*, *P. maculatus*, and multiple other bamboo leaf varieties, in which apigenin and luteolin glycosides are repeatedly prominent [7,8,9,10]. The number of annotations in the present fraction (31) exceeds the 13 compounds reported for *P. edulis* and the 22 reported for *B. chungii* and equals the 31 reported for *P. maculatus* [7,8,9]. These numerical differences must not be interpreted as direct evidence of species-level chemical richness because extraction procedures, enrichment steps, chromatographic methods, detection thresholds, environmental conditions, and growth stage differ among studies [11,12,13,27,28]. Targeted determination of seven flavonoids in bamboo-leaf extracts has also been reported [29], but those quantitative results cannot be compared with the within-spectrum relative ion intensities reported here. The present contribution is therefore a species- and fraction-specific qualitative dataset rather than a comparative quantitative survey.

The seven compounds matched to in-house reference standards satisfy MSI Level 1 criteria because retention time and MS/MS behavior were compared under the same analytical conditions [20]. For the remaining twenty-four features, accurate mass, aglycone-related ions, sugar losses, and literature comparisons support compound-class annotations, but structurally similar positional isomers often yield closely related tandem spectra. These compounds are consequently reported at MSI Level 3, and exact glycosylation positions have been removed from the revised names unless supported by a reference standard. Isolation followed by NMR analysis or comparison with additional authentic standards would be required for definitive positional assignments.

The sample-preparation workflow also defines the scope of the findings. Petroleum-ether partitioning and AB-8 adsorption followed by collection of the 40% ethanol fraction selectively enriched certain constituents and may have excluded or underrepresented others. Accordingly, the annotations describe this flavonoid-enriched fraction and not the comprehensive flavonoid profile of intact *B. textilis* leaves. In addition, the spectral relative ion intensities are not quantitative estimates of compound abundance, and formal chromatographic resolution data were not available to exclude all co-elution. Despite these limitations, the compiled retention times, accurate precursor masses, and diagnostic product ions provide a traceable reference for future confirmation and qualitative comparison of bamboo flavonoid glycosides.

## 4. Materials and Methods

### 4.1. Plant Material, Reagents, and Reference Standards

Fresh leaves of *Bambusa textilis* (200 g) were collected in November 2019 from the bamboo garden of the Jiangxi Academy of Forestry, Nanchang, Jiangxi Province, China. The plant material was authenticated by Fuming Xiao of the Jiangxi Academy of Forestry. The seven reference standards listed in Table 2 were not purchased commercially; they had been isolated in-house from bamboo leaves. Their structures were established by MS and NMR using an Agilent 6520 Q-TOF mass spectrometer (Santa Clara, CA, USA) and a Bruker 300 NMR spectrometer (Fällanden, Switzerland), and their purities were greater than 95% as determined by HPLC-PDA using a Waters 2695 system equipped with a 2996 PDA detector (Milford, MA, USA). The HPLC-PDA and NMR instruments were used for characterization of the reference standards rather than for profiling the *B. textilis* fraction.

LC/MS-grade methanol and acetonitrile were purchased from Merck (Darmstadt, Germany). MS-grade ammonium formate was obtained from Sigma (St. Louis, MO, USA). Commercial ultrapure water from Watsons (Hong Kong, China) was used to prepare the aqueous mobile phase and other LC-MS solutions. The 0.2 µm polytetrafluoroethylene syringe filters were obtained from Whatman (Maidstone, UK). Ethanol (95%) and petroleum ether were analytical grade and used for sample preparation. AB-8 macroporous adsorption resin was supplied by Anhui Sanxing Resin Technology Co., Ltd. (Bengbu, Anhui, China).

### 4.2. Instruments

The main instruments used in this study are listed in Table 3. The HPLC-PDA and NMR systems were used solely for purity assessment and structural characterization of the in-house reference standards, respectively.

### 4.3. Extraction, Purification, and Sample Preparation

Fresh leaves were stripped from the petioles, dried naturally at room temperature (20–25 °C), and milled to approximately 40 mesh. Leaf powder (50 g) was refluxed three times with 95% ethanol at a solid-to-liquid ratio of 1:10 (m/v) for 6 h at 60 °C. The combined extracts were filtered and concentrated under reduced pressure. The residue was suspended in water and extracted three times with petroleum ether at a water-to-petroleum-ether volume ratio of 1:3. The aqueous phase was concentrated to remove water and purified on a column packed with AB-8 macroporous adsorption resin (Anhui Sanxing Resin Technology Co., Ltd., Bengbu, Anhui, China) by sequential elution with water, 20% ethanol, 40% ethanol, and 100% ethanol. The 40% ethanol fraction was collected, concentrated, and freeze-dried to obtain the flavonoid-enriched *B. textilis* leaf fraction analyzed in this study.

A 0.05 g portion of the dried fraction was dissolved in LC/MS-grade methanol to 5 mg/mL and sonicated for 15 min. The supernatant was passed through a 0.2 µm PTFE syringe filter (Whatman, UK) and transferred to an autosampler vial for UHPLC-Q-TOF-MS analysis. Each reference standard was prepared in LC/MS-grade methanol at 0.02 mg/mL, sonicated for 15 min, and filtered through the same type of 0.2 µm PTFE membrane. The injection volume was 10 µL.

### 4.4. UHPLC Conditions

Chromatographic analysis was performed with an Agilent 1200 UHPLC system coupled to an Agilent 6520 high-resolution Q-TOF mass spectrometer. The UHPLC conditions are summarized in Table 4. The PDA acquisition range was 190–400 nm, and the chromatogram was monitored at 350 nm because conjugated flavone glycosides show strong long-wavelength absorption in this region and because the same wavelength has been widely used for bamboo-flavonoid profiling [7,8,9,10]. The UV trace was used for chromatographic visualization and not for quantification or definitive structural assignment.

### 4.5. Q-TOF-MS Conditions

The Q-TOF mass spectrometer was equipped with an electrospray ionization source and operated in negative-ion mode. The operating conditions are listed in Table 5. Collision energy was calculated using the instrument function CE = 4 × (*m*/*z*)/100 + 8 eV.

### 4.6. Fragment-Ion Nomenclature

**Figure 12:**
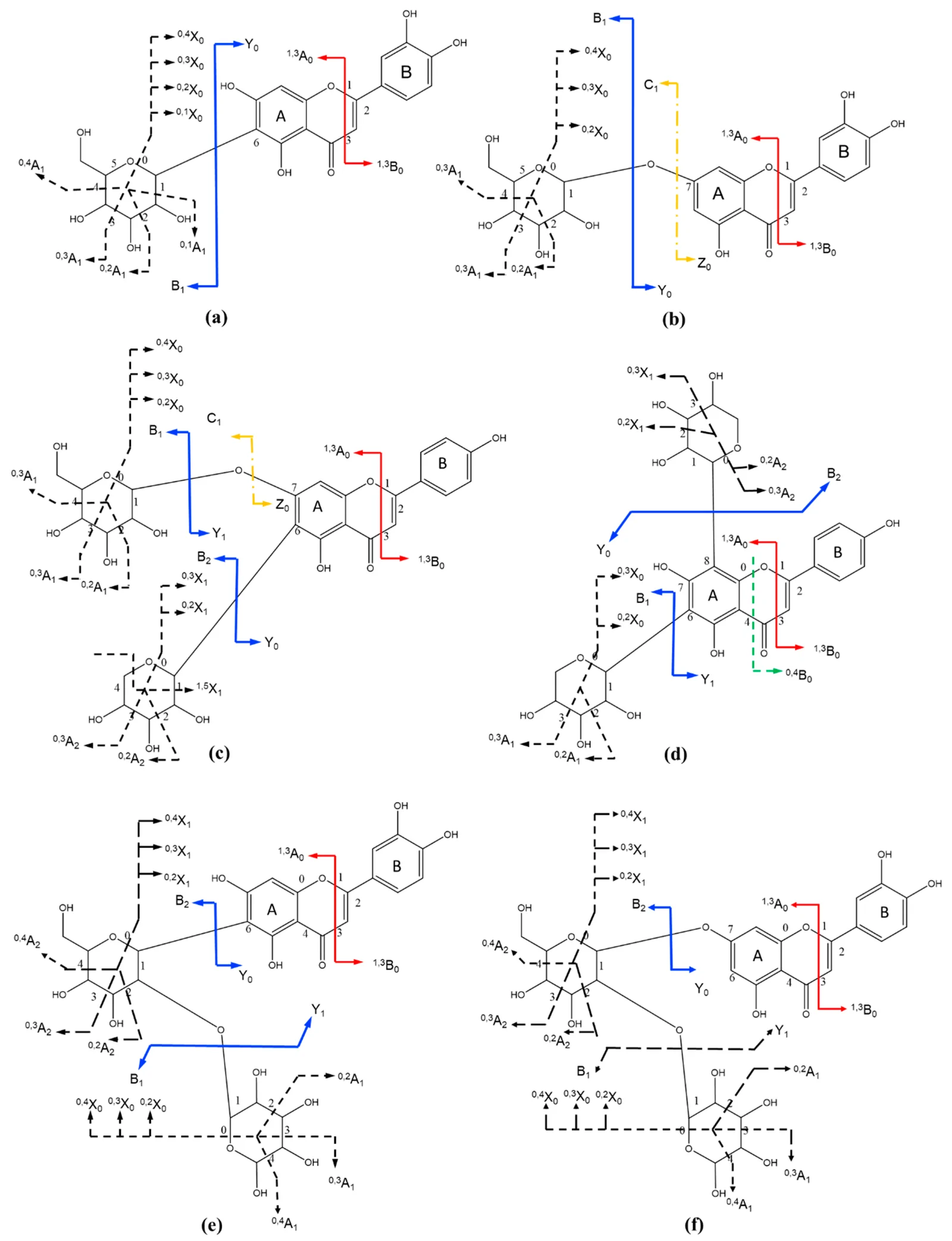
(**a**–**f**) Fragment-ion nomenclature used for flavone O,O-diglycosides.

Fragment ions were assigned according to the carbohydrate nomenclature of Domon and Costello and the flavonoid fragmentation nomenclature described by Ma et al. [18,19]. The notation applied to flavone O,O-diglycosides is illustrated in Figure 12.

## 5. Conclusions

UHPLC-ESI-Q-TOF-MS/MS in negative-ion mode was used to characterize flavonoid glycosides in a 40% ethanol, resin-enriched fraction from *Bambusa textilis* leaves. Seven compounds were identified at MSI Level 1 through comparison with authentic in-house standards, and twenty-four features were conservatively annotated at MSI Level 3 from accurate-mass and MS/MS evidence. The annotations encompassed C-glycosides, O-glycosides, di-C-glycosides, di-C,O-glycosides, C,O-diglycosides, and an O,O-diglycoside. The reported retention times and diagnostic product ions provide reference data for future comparison and structural confirmation of bamboo flavonoid glycosides. These findings apply only to the 40% ethanol, resin-enriched fraction and should not be interpreted as a complete leaf metabolome or quantitative composition. Definitive positional assignments for compounds lacking standards require isolation and NMR analysis or comparison with additional authentic standards.

## Figures and Tables

**Table 1:** Flavonoid-related constituents identified or tentatively annotated in the *Bambusa textilis* leaf fraction by UHPLC-ESI-Q-TOF-MS/MS.

No.	tR (min)	Formula	Calculated/Measured [M-H]^−^ *m*/*z* (Relative Ion Intensity, %)	Mass Error (ppm)	Product Ions, *m*/*z* (Relative Ion Intensity, %)	Tentative Annotation/MSI Confidence Level
1	13.37	C_27_H_30_O_15_	593.1512/593.1501 (40.23)	−1.84	503.1150 (0.88), 473.1100 (9.69), 431.0974 (17.45), 413.0902 (1.47), 341.0674 (10.95), 311.0555 (100), 297.0398 (40.13), 281.0424 (2.71), 269.0472 (3.4), 117.0352 (1)	Apigenin di-C-glucoside positional isomer (MSI Level 3)
2	13.75	C_26_H_28_O_15_	579.1355/579.1361 (7.41)	+0.96	459.0938 (72.21), 447.0971 (2.22), 429.0835 (17.23), 357.0610 (35.46), 339.0520 (19.57), 327.0516 (47.52), 309.0410 (35.96), 298.0491 (100), 285.0402 (13.81), 133.0282 (1.55)	Luteolin C-glucosyl-O-arabinoside positional isomer (MSI Level 3)
3	14.36	C_26_H_28_O_14_	563.1406/563.1418 (30.82)	+2.08	473.1109 (34.1), 443.0998 (53.74), 413.0891 (11.74), 383.0794 (75.43), 353.0688 (100), 323.0589 (3.67), 117.0328 (1.53)	Apigenin C-arabinosyl-C-glucoside positional isomer (MSI Level 3)
4	14.94	C_21_H_20_O_11_	447.0933/447.0914 (0.64)	−4.22	429.0816 (2.3), 357.0624 (71.19), 339.0503 (11.61), 327.0511 (100), 297.0407 (29.31), 285.0412 (21.46), 133.0308 (4.04)	Luteolin 6-C-glucoside (MSI Level 1)
5	15.71	C_27_H_30_O_15_	593.1512/593.1518 (7.74)	+1.02	473.1096 (75.36), 447.0918 (1.32), 429.0836 (20.22), 357.0608 (35.04), 339.0511 (20.67), 327.0520 (57.53), 309.0416 (37.58), 298.0492 (100), 285.0415 (13.41), 133.0294 (1.13)	Luteolin 6-C-(2″-O-rhamnosyl)glucoside (MSI Level 1)
6	19.14	C_27_H_30_O_15_	593.1512/593.1529 (2.44)	+2.88	473.1114 (5.04), 431.0939 (1.98), 413.0867 (20.99), 341.0675 (5.35), 323.0533 (10.65), 311.0578 (11.81), 293.0454 (100), 269.0463 (5.78)	Apigenin C-glucosyl-O-glucoside positional isomer (MSI Level 3)
7	19.54	C_26_H_28_O_14_	563.1406/563.1409 (48.75)	+0.48	473.1056 (19.43), 401.0883 (59.5), 383.1164 (12.55), 341.0687 (27.39), 311.0575 (100), 297.0440 (42.17), 269.0450 (15.48)	Apigenin 6-C-arabinoside 7-O-glucoside (MSI Level 1)
8	20.68	C_26_H_28_O_14_	563.1406/563.1384 (1.13)	−3.96	443.0976 (3.37), 413.0874 (16.05), 353.0663 (2.98), 341.0678 (6.05), 323.0561 (6.28), 311.0547 (9.48), 293.0454 (100), 269.0419 (2.56), 117.0359 (0.41)	Apigenin C-glucosyl-O-arabinoside positional isomer (MSI Level 3)
9	21.93	C_21_H_22_O_11_	449.1089/449.1064 (0.61)	−5.64	287.0571 (15.93), 151.0028 (100), 135.0440 (38.86)	Eriodictyol O-glucoside positional isomer (MSI Level 3)
10	22.32	C_21_H_20_O_10_	431.0984/431.0974 (0.51)	−2.25	413.0818 (0.49), 341.0668 (34.2), 323.0554 (10.35), 311.0556 (100), 281.0448 (6.75), 269.0460 (7.08), 151.0045 (0.34), 117.0339 (1.78)	Apigenin 6-C-glucoside (MSI Level 1)
11	22.96	C_27_H_30_O_14_	577.1563/577.1579 (2.23)	+2.81	457.1151 (5.11), 431.0954 (0.53), 413.0879 (17.03), 353.0669 (4.38), 341.0673 (11.33), 323.0566 (10.08), 311.0557 (13.81), 293.0554 (100), 269.0445 (4.01)	Apigenin C-glucosyl-O-rhamnoside positional isomer (MSI Level 3)
12	24.55	C_25_H_26_O_13_	533.1301/533.1301 (34.77)	+0.07	473.1072 (33.48), 443.0983 (53), 413.0878 (14.61), 383.0781 (100), 353.0767 (95.74), 117.0339 (1.49)	Apigenin 6,8-di-C-arabinoside (MSI Level 1)
13	25.31	C_21_H_20_O_11_	447.0933/447.0914 (5.15)	−4.22	285.0391 (100), 284.0319 (36.58), 151.0041 (3.16), 133.0323 (1.64)	Luteolin 7-O-glucoside (MSI Level 1)
14	25.54	C_27_H_30_O_15_	593.1512/593.1532 (10.18)	+3.38	447.0948 (0.69), 285.0406 (100), 151.0355 (0.54), 133.0295 (0.45)	Luteolin O-glucosyl-O-rhamnoside positional isomer (MSI Level 3)
15	27.46	C_27_H_30_O_14_	577.1563/577.1551 (52.27)	−2.04	473.1135 (9.98), 415.1026 (39.58), 397.0800 (4.8), 353.0615 (10.89), 341.0621 (26.14), 311.0548 (100), 285.0426 (38.6)	Luteolin C-boivinosyl-O-glucoside positional isomer I (MSI Level 3)
16	28.43	C_27_H_30_O_14_	577.1563/577.1545 (22.84)	−3.08	473.1076 (8.29), 415.1038 (100), 371.0768 (7.95), 353.0662 (54.42), 341.0655 (8.04), 311.0568 (67.09), 285.0389 (3.67), 133.0306 (1.41)	Luteolin C-boivinosyl-O-glucoside positional isomer II (MSI Level 3)
17	31.32	C_25_H_26_O_13_	533.1301/533.1287 (29.17)	−2.56	473.1020 (20.05), 443.1016 (59.82), 413.0841 (11.27), 383.0764 (85.28), 353.0655 (70.37)	Apigenin di-C-arabinoside positional isomer (MSI Level 3)
18	31.86	C_26_H_28_O_14_	563.1406/563.1404 (19.16)	−0.41	473.1090 (51.44), 417.0855 (5.64), 399.0720 (31.14), 357.0605 (37.38), 339.0520 (11.58), 327.0510 (51.9), 309.0410 (19.71), 298.0495 (100), 285.0410 (16.82)	Luteolin C-arabinosyl-O-rhamnoside positional isomer (MSI Level 3)
19	35.10	C_26_H_28_O_14_	563.1406/563.1425 (19.16)	+3.32	459.0935 (72.59), 431.0999 (5.23), 413.0851 (21.59), 357.0618 (22.55), 339.0468 (17.4), 327.0491 (35.67), 309.0411 (21.18), 298.0480 (100), 285.0409 (13.23)	Luteolin C-rhamnosyl-O-arabinoside positional isomer (MSI Level 3)
20	37.52	C_27_H_30_O_13_	561.1614/561.1615 (93.88)	+0.24	457.1135 (70.41), 399.1067 (28.27), 381.0975 (10.99), 355.0916 (4.53), 337.0741 (37.44), 295.0605 (100), 281.0470 (6.12), 269.0457 (8.12)	Apigenin C-boivinosyl-O-glucoside positional isomer I (MSI Level 3)
21	39.04	C_27_H_30_O_13_	561.1614/561.1599 (71.01)	−2.61	457.1105 (56.28), 399.1070 (41.46), 381.0977 (9.9), 355.0816 (2.6), 337.0723 (19.65), 295.0616 (100), 281.0469 (4.83), 269.0428 (8.45)	Apigenin C-boivinosyl-O-glucoside positional isomer II (MSI Level 3)
22	39.93	C_27_H_30_O_14_	577.1563/577.1580 (8.55)	+2.98	473.1081 (69.07), 431.0935 (5.26), 413.0858 (17.49), 357.0624 (28.97), 339.0504 (13.95), 327.0505 (48.56), 309.0402 (21.62), 298.0484 (100), 285.0410 (11.14)	Luteolin C-rhamnosyl-O-rhamnoside positional isomer I (MSI Level 3)
23	42.59	C_26_H_28_O_13_	547.1457/547.1452 (7.78)	−0.94	457.1142 (11.09), 401.0869 (2.81), 383.0767 (71.52), 341.0668 (34.13), 323.0564 (18.66), 311.0555 (22.92), 293.0457 (79.32), 269.0464 (10.98)	Apigenin C-arabinosyl-O-rhamnoside positional isomer (MSI Level 3)
24	43.18	C_23_H_24_O_12_	491.1195/491.1179 (9.38)	−3.26	476.0982 (67.15), 461.0745 (79.44), 343.0459 (19.89), 329.0680 (20.89), 313.0362 (100), 299.0208 (30.39), 285.0384 (15.46), 271.0234 (16.82)	Tricin 7-O-glucoside (MSI Level 1)
25	45.34	C_27_H_28_O_14_	575.1406/575.1388 (5.21)	−3.18	473.1096 (9.53), 429.0827 (10.25), 411.0716 (25.46), 385.0599 (19.93), 367.0459 (56.53), 325.0353 (100), 298.0479 (68.82), 285.0397 (26.29), 133.0276 (2.39)	Cassiaoccidentalin B-type isomer (MSI Level 3)
26	47.91	C_23_H_24_O_12_	491.1195/491.1155 (8.94)	−8.14	371.0700 (2.19), 329.0665 (71.56), 313.0348 (100)	Tricin O-glucoside positional isomer (MSI Level 3)
27	48.47	C_27_H_30_O_13_	561.1614/561.1604 (68.76)	−1.72	457.1105 (42.7), 399.1085 (43.89), 381.1070 (6.56), 355.0986 (4.18), 337.0751 (41.98), 295.0614 (100), 281.0425 (5.97), 269.0565 (8.6)	Apigenin C-boivinosyl-O-glucoside positional isomer III (MSI Level 3)
28	54.17	C_27_H_30_O_14_	577.1563/577.1540 (16.17)	−3.95	473.1082 (68.74), 431.0996 (5.74), 413.0879 (26.52), 357.0610 (36.54), 339.0514 (17.33), 327.0499 (57.07), 309.0412 (21.63), 298.0469 (100), 285.0387 (14.03)	Luteolin C-rhamnosyl-O-rhamnoside positional isomer II (MSI Level 3)
29	54.81	C_27_H_30_O_13_	561.1614/561.1620 (4.42)	+1.13	415.0991 (2.03), 397.0930 (45.64), 341.0665 (20.95), 323.0549 (17.84), 311.0567 (29.81), 293.0445 (77.27), 269.0450 (11.58), 117.0352 (2.29)	Apigenin C-rhamnosyl-O-rhamnoside positional isomer I (MSI Level 3)
30	59.35	C_27_H_28_O_13_	559.1457/559.1436 (2.11)	−3.78	457.1121 (2.18), 413.0863 (3.1), 395.0742 (27.14), 351.0695 (11.39), 321.0408 (73.61), 269.0452 (39.54)	Cassiaoccidentalin A-type isomer (MSI Level 3)
31	65.80	C_27_H_30_O_13_	561.1614/561.1609 (8.13)	−0.83	415.1060 (1.42), 397.0929 (57.45), 341.0663 (14.99), 323.0562 (13.21), 311.0577 (14.52), 293.0452 (97.54), 269.0478 (8), 117.0360 (0.77)	Apigenin C-rhamnosyl-O-rhamnoside positional isomer II (MSI Level 3)

Mass error was calculated as (measured *m*/*z* − calculated *m*/*z*)/calculated *m*/*z* × 10^6^. Values in parentheses following precursor and product ions are instrument-reported relative ion intensities; product-ion values are normalized to the base peak (100%) within each MS/MS spectrum. These values are not chromatographic peak areas or estimates of compound concentration. MSI Level 1 denotes confirmation against an authentic reference standard analyzed under the same conditions; MSI Level 3 denotes a putatively characterized compound or compound class for which the exact positional structure remains unresolved.

**Table 2:** In-house reference standards used for UHPLC-ESI-Q-TOF-MS/MS analysis.

Reference Standard	Source	Purity
Luteolin 6-C-glucoside	Isolated in-house from bamboo leaves	>95% by HPLC-PDA
Apigenin 6-C-glucoside	Isolated in-house from bamboo leaves	>95% by HPLC-PDA
Luteolin 7-O-glucoside	Isolated in-house from bamboo leaves	>95% by HPLC-PDA
Tricin 7-O-glucoside	Isolated in-house from bamboo leaves	>95% by HPLC-PDA
Apigenin 6,8-di-C-arabinoside	Isolated in-house from bamboo leaves	>95% by HPLC-PDA
Apigenin 6-C-arabinoside 7-O-glucoside	Isolated in-house from bamboo leaves	>95% by HPLC-PDA
Luteolin 6-C-(2″-O-rhamnosyl)glucoside	Isolated in-house from bamboo leaves	>95% by HPLC-PDA

**Table 3:** Main instruments and their roles in the study.

Instrument	Model/Manufacturer
Analytical balance	BP221S (Sartorius, Göttingen, Germany)
Rotary evaporator	EYELA N-1000 (Tokyo Rikakikai Co., Ltd., Tokyo, Japan)
Ultrasonic cleaner	KQ-250B (Kunshan Ultrasonic Instruments, Kunshan, China)
UHPLC system	Agilent 1200 (Agilent Technologies, Singapore)
High-resolution Q-TOF mass spectrometer	Agilent 6520 (Singapore)
HPLC-PDA system	Waters 2695 with 2996 PDA detector (USA); reference-standard purity assessment
NMR spectrometer	Bruker 300 NMR (Switzerland); reference-standard structural characterization

**Table 4:** UHPLC conditions.

Parameter	Setting
Column	Agilent Zorbax RRHD SB-C18 (150 × 2.1 mm, 1.8 µm)
Mobile phase	A, 10 mmol/L ammonium formate in water; B, acetonitrile
Gradient	0–30 min, 10–15% B; 30–40 min, 15% B; 40–60 min, 15–20% B; 60–80 min, 20–30% B
Column temperature	40 °C
Flow rate	0.25 mL/min
Injection volume	10 µL
PDA acquisition range/monitoring wavelength	190–400 nm/350 nm

**Table 5:** Q-TOF-MS conditions.

Parameter	Setting
Ionization mode	Negative
Gas temperature	350 °C
Drying-gas flow	8 L/min
Nebulizer pressure	35 psig
Sheath-gas flow/temperature	11 L/min; 350 °C
Capillary/nozzle voltage	3500 V; 1000 V
Fragmentor/skimmer voltage	175 V; 65 V
Octopole RF voltage	750 V
Collision gas	N_2_
MS scan range	*m*/*z* 50–1100
MS/MS scan range	*m*/*z* 50–800

## Data Availability

The original contributions presented in this study are included in the article. Further inquiries can be directed to the corresponding author.

## References

[1] Nirmala C., Bisht M.S., Bajwa H.K., Santosh O. (2018). Bamboo: A rich source of natural antioxidants and its applications in the food and pharmaceutical industry. Trends Food Sci. Technol..

[2] He Y., Yue Y. (2008). A review of the effective components and applications of extracts from bamboo leaves. Biomass Chem. Eng..

[3] Lu B., Wu X., Shi J., Dong Y., Zhang Y. (2006). Toxicology and safety of antioxidant of bamboo leaves. Part 2: Developmental toxicity test in rats with antioxidant of bamboo leaves. Food Chem. Toxicol..

[4] Wang J., Yue Y.D., Tang F., Sun J. (2012). TLC screening for antioxidant activity of extracts from fifteen bamboo species and identification of antioxidant flavone glycosides from leaves of *Bambusa textilis* McClure. Molecules.

[5] Wang J. (2012). Screening and Determination of Active Components and Analysis of Volatile Compounds from Bamboo Leaves of the Genus Bambusa. Doctoral Dissertation.

[6] Sun G. (2010). Studies on Chemical Constituents and Biological Activities of *Bambusa pervariabilis* McClure Leaves. Doctoral Dissertation.

[7] Shao S.-Y., Ting Y., Wang J., Sun J., Guo X.-F. (2020). Characterization and identification of the major flavonoids in *Phyllostachys edulis* leaf extract by UPLC-QTOF-MS/MS. Acta Chromatogr..

[8] Yuan T., Guo X.-F., Shao S.-Y., An R.-M., Wang J., Sun J. (2021). Characterization and identification of flavonoids from *Bambusa chungii* leaves extract by UPLC-ESI-Q-TOF-MS/MS. Acta Chromatogr..

[9] Shao S.-Y., Wang J., Yao X., Xun H., Guo X.-F. (2024). Characterization and identification of major flavonoids of bamboo leaf extract by HPLC/ESI-QTOF-MS/MS. J. Asian Nat. Prod. Res..

[10] Farias C.A.A., dos Reis A.R., de Morais D.R., Camponogara J.A., Bettio L., Pudenzi M.A., Ballus C.A., Barcia M.T. (2024). Phenolic diversity and antioxidant potential of different varieties of bamboo leaves using LC-ESI-QTOF-MS/MS and LC-ESI-QqQ-MS/MS. Food Res. Int..

[11] Chitiva L.C., Lozano-Puentes H.S., Londono X., Leao T.F., Cala M.P., Ruiz-Sanchez E., Diaz-Ariza L.A., Prieto-Rodriguez J.A., Castro-Gamboa I., Costa G.M. (2023). Untargeted metabolomics approach and molecular networking analysis reveal changes in chemical composition under the influence of altitudinal variation in bamboo species. Front. Mol. Biosci..

[12] Cheng Y., Wan S., Yao L., Ding L., Wu T., Chen Y., Zhang A., Lu C. (2023). Bamboo leaf: A review of traditional medicinal property, phytochemistry, pharmacology, and purification technology. J. Ethnopharmacol..

[13] Shen H., Wang Y., Shi P., Li H., Chen Y., Hu T., Yu Y., Wang J., Yang F., Luo H. (2024). Effects of the species and growth stage on the antioxidant and antifungal capacities, polyphenol contents, and volatile profiles of bamboo leaves. Foods.

[14] Sun S., Liu M., He J., Li K., Zhang X., Yin G. (2019). Identification and determination of seven phenolic acids in Brazilian green propolis by UPLC-ESI-QTOF-MS and HPLC. Molecules.

[15] Quirantes-Pine R., Lozano-Sanchez J., Herrero M., Ibanez E., Segura-Carretero A., Fernandez-Gutierrez A. (2013). HPLC-ESI-QTOF-MS as a powerful analytical tool for characterizing phenolic compounds in olive leaf extracts. Phytochem. Anal..

[16] Yang S.T., Wu X., Rui W., Guo J., Feng Y.F. (2015). UPLC/Q-TOF-MS analysis for identification of hydrophilic phenolics and lipophilic diterpenoids from Radix *Salviae miltiorrhizae*. Acta Chromatogr..

[17] Yan M., Chen M., Zhou F., Cai D., Bai H., Wang P., Lei H., Ma Q. (2019). Separation and analysis of flavonoid chemical constituents in flowers of Juglans regia L. by ultra-high-performance liquid chromatography-hybrid quadrupole time-of-flight mass spectrometry. J. Pharm. Biomed. Anal..

[18] Domon B., Costello C.E. (1988). A systematic nomenclature for carbohydrate fragmentations in FAB-MS/MS spectra of glycoconjugates. Glycoconj. J..

[19] Ma Y.L., Li Q.M., Van den Heuvel H., Claeys M. (1997). Characterization of flavone and flavonol aglycones by collision-induced dissociation tandem mass spectrometry. Rapid Commun. Mass Spectrom..

[20] Sumner L.W., Amberg A., Barrett D., Beale M.H., Beger R., Daykin C.A., Fan T.W.-M., Fiehn O., Goodacre R., Griffin J.L. (2007). Proposed minimum reporting standards for chemical analysis: Chemical Analysis Working Group (CAWG) Metabolomics Standards Initiative (MSI). Metabolomics.

[21] Singh A., Kumar S., Bajpai V., Reddy T.J., Rameshkumar K.B., Kumar B. (2015). Structural characterization of flavonoid C- and O-glycosides in an extract of *Adhatoda vasica* leaves by liquid chromatography with quadrupole time-of-flight mass spectrometry. Rapid Commun. Mass Spectrom..

[22] Ablajan K., Abliz Z., Shang X.Y., He J.M., Zhang R.P., Shi J.G. (2006). Structural characterization of flavonol 3,7-di-O-glycosides and determination of the glycosylation position by using negative-ion electrospray ionization tandem mass spectrometry. J. Mass Spectrom..

[23] Hasegawa T., Tanaka A., Hosoda A., Takano F., Ohta T. (2008). Antioxidant C-glycosyl flavones from the leaves of *Sasa kurilensis* var. gigantea. Phytochemistry.

[24] Wolff T., Berrueta L.A., Valente L.M.M., Barboza R.S., Neris R.L.S., Guimaraes-Andrade I.P., Assuncao-Miranda I., Nascimento A.C., Gomes M., Gallo B. (2019). Comprehensive characterization of polyphenols in leaves and stems of three anti-dengue virus type-2 active Brazilian *Faramea* species (Rubiaceae) by HPLC-DAD-ESI-MS/MS. Phytochem. Anal..

[25] Figueirinha A., Paranhos A., Perez-Alonso J.J., Santos-Buelga C., Batista M.T. (2008). *Cymbopogon citratus* leaves: Characterization of flavonoids by HPLC-PDA-ESI/MS/MS and an approach to their potential as a source of bioactive polyphenols. Food Chem..

[26] Perez A.J., Hussain S.M., Pecio L., Kowalczyk M., Herling V.R., Stochmal A. (2016). UHPLC-HR-QTOF-MS-based metabolomics reveals key differences between *Brachiaria decumbens* and *B. brizantha*, two similar pastures with different toxicities. J. Agric. Food Chem..

[27] Benjamin M.A.Z., Ng S.-Y., Saikim F.H., Rusdi N.A. (2022). The effects of drying techniques on phytochemical contents and biological activities on selected bamboo leaves. Molecules.

[28] Chitiva L.C., Rezende-Teixeira P., Leao T., Lozano-Puentes H.S., Londono X., Diaz-Ariza L.A., Costa-Lotufo L.V., Prieto-Rodriguez J.A., Costa G.M., Castro-Gamboa I. (2024). Metabolomic profiling of *Guadua* species and its correlation with antioxidant and cytotoxic activities. ACS Omega.

[29] Gu L.-L., Yao X., An R.-M., Guo X.-F. (2024). Determination and antioxidant analysis of seven flavonoids in bamboo-leaf extracts. Chin. J. Chromatogr..

